# Do You See what I See? Tracking the Perceptual Beliefs of Robots

**DOI:** 10.1016/j.isci.2020.101625

**Published:** 2020-09-29

**Authors:** Sam Thellman, Tom Ziemke

**Affiliations:** 1 Department of Computer and Information Science, Cognition & Interaction Lab, Linköping University, Linköping 581 83, Sweden

**Keywords:** Human-Computer Interaction, Psychology, Research Methodology Social Sciences

## Abstract

Keeping track of others' perceptual beliefs—what they perceive and know about the current situation—is imperative in many social contexts. In a series of experiments, we set out to investigate people's ability to keep track of what robots know or believe about objects and events in the environment. To this end, we subjected 155 experimental participants to an anticipatory-looking false-belief task where they had to reason about a robot's perceptual capability in order to predict its behavior. We conclude that (1) it is difficult for people to track the perceptual beliefs of a robot whose perceptual capability potentially differs significantly from human perception, (2) people can gradually “tune in” to the unique perceptual capabilities of a robot over time by observing it interact with the environment, and (3) providing people with verbal information about a robot's perceptual capability might not significantly help them predict its behavior.

## Highlights

• Keeping track of what others perceive and know is important in social interaction
• It is difficult to track the perceptual beliefs of robots with non-human perception
• People can learn from observation about robots' perceptual capabilities
• Verbal information about robots' capabilities might not facilitate belief tracking

## Graphical Abstract

**Figure:**
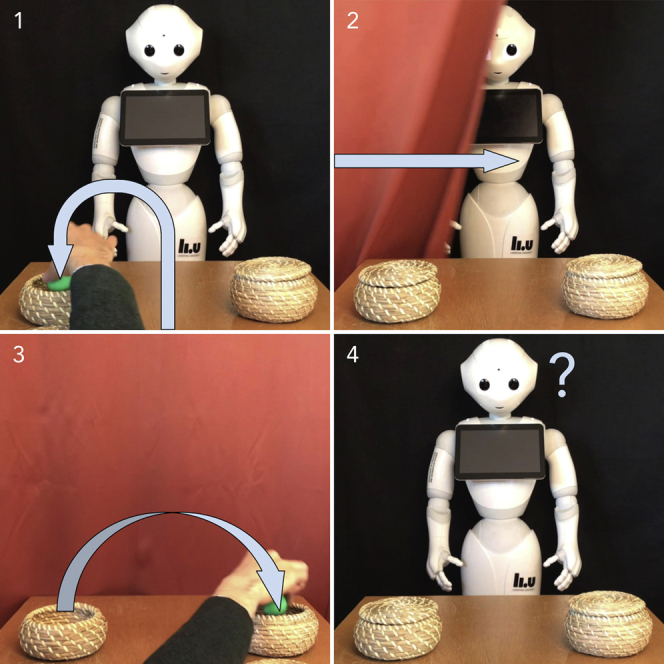

## Introduction

Failure to grasp what others know and believe about objects and events in the environment can give rise to a host of communicative and interactive issues, including difficulties in predicting behavior (Dennett, 1989; Kovács et al., 2010; Wimmer and Perner, 1983), communicating verbally (Clark and Marshall, 1981; Krauss and Fussell, 1991; Nickerson, 1999), and deciding on one's own future states and actions (FeldmanHall and Shenhav, 2019). We have suggested elsewhere that perceptual belief tracking might be particularly problematic in the context of human-robot interactions, where the perceptual capabilities of robots are diverse and often difficult to infer (Thellman and Ziemke, n.d., submitted), which limits understandability (Hellström and Bensch, 2018; Ziemke, 2020) or explainability (de Graaf et al., 2018). This problem, which we call the *perceptual belief problem* in human-robot interaction (HRI), has so far not been studied empirically to any significant extent. In the reported study we therefore investigated people's ability to track the perceptual beliefs of robots in a series of four experiments. In the first pair of experiments, we focused on advancing our understanding of the problem by asking whether it is in fact more difficult to infer the perceptual beliefs of a robot with non-human vision as compared with human vision (*Experiment 1: Visual Belief Tracking*) and hearing (*Experiment 2: Auditory Belief Tracking*). In the second pair of experiments we explored different types of solutions to the problem by investigating if people are able to learn or “tune in” to the non-human perception of a robot by observing it interact with the environment (*Experiment 3: Endogenous Adaptation*) and whether the process of inferring a robot's perceptual beliefs can be facilitated by providing people with verbal information about its perceptual capability (*Experiment 4: Exogenous Facilitation*).

The presented work is part of our more general, long-term endeavor to explore the characteristics and limitations of people's ability to predict robot behavior based on intentional folk-psychological constructs (Thellman et al., 2017; Thellman and Ziemke, 2019; Ziemke, 2020). People rely heavily on ascribing intentional states (e.g., beliefs and desires) to predict and explain the behavior of themselves and others in social interactions (Dennett, 1989; Fodor, 1987; Heider, 1958; Malle, 2006). Dennett (1989) referred to this as “taking the intentional stance.” The intentional stance may play an important role also in interactions with artificial systems (Dennett, 1989; McCarthy, 1979), including robots (Marchesi et al., 2019; Thellman et al., 2017; Thill and Ziemke, 2017; Wiese et al., 2017). Recent efforts have been made toward studying mental state ascription to robots empirically. However, most research in this area has focused on understanding what kinds of mental states people ascribe to robots (e.g., de Graaf and Malle, 2019; Fiala et al., 2014; Gray et al., 2007; Malle, 2019; Petrovych et al., 2018; Thellman et al., 2017; Waytz et al., 2014; Zhao et al., 2016). That means, there is so far hardly any research addressing the call for research made by Lee et al. (2005), to “study how people's mental models affect how they actually interact with robots.” This is problematic because efforts to design and improve human-robot interactions need to be informed by knowledge about the effects that people's (mis-) understandings of robots have on human-robot interactions. The present study addresses this by studying assumptions about robots that have direct consequences for interactions with robots. Some beliefs about robots—particularly beliefs about *their* beliefs (Dennett, 1989)—give rise to predictions about how they are going to behave in the future. For example, the belief that a robot knows (or does not know) about objects, such as oneself, moving in its environment, may yield the prediction that the robot will (or will not) act to avoid those objects in a situation where there is risk for collision. Despite a growing interest in the role of mental state attribution in people's mental models of robots, and the importance of perceptual belief tracking in the context of social interaction, no research has so far targeted people's ability to predict the behavior of robots based on assumptions about how they perceive the environment. The aim of the study presented here is to contribute toward filling this gap of research.

## Results

### Experiment 1: Visual Belief Tracking

**Figure 1:**
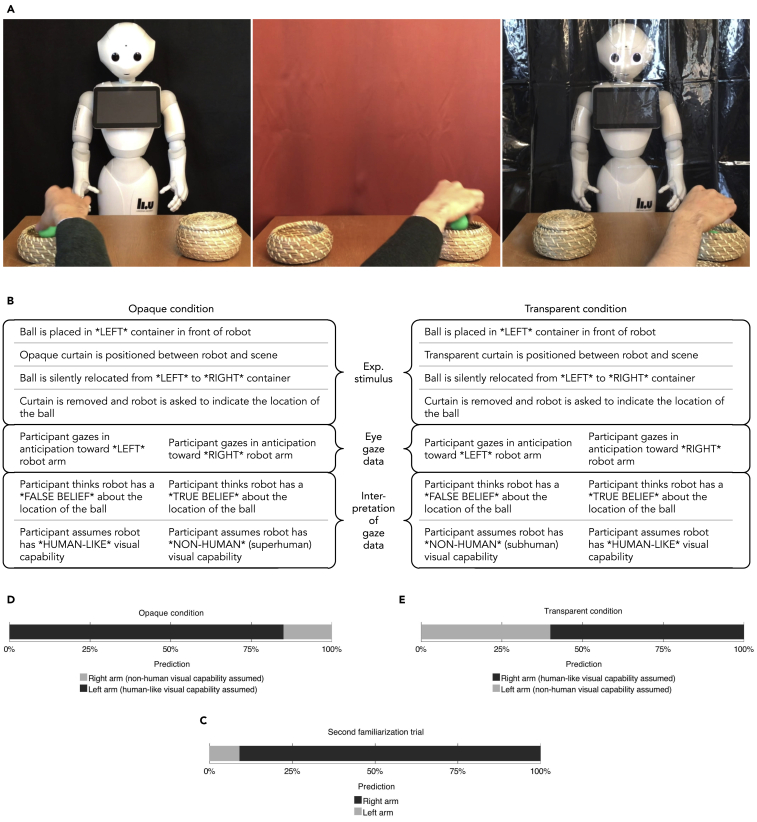
Visual Belief Tracking Experiment (A) Participants in experiment 1 watched a video of a ball being placed in one of two baskets in front of a robot (left). An opaque or transparent curtain was pulled in front of the robot (middle and right, respectively), and the ball was silently removed from the basket and placed into the other basket. The participant now had to judge whether the robot could see the displacement of the ball through the curtain in order to predict which basket the robot will point to when asked about the ball's location. This judgment was inferred based on the presence of anticipatory saccades toward the robot's arms that were measured using eye-tracking technology. (B) Overview of the logic of the experiment. ∗LEFT∗ and ∗RIGHT∗ refer to baskets and robot arms from the reader's perspective. (C) The proportion of left and right arm predictions in the second familiarization trial. (D) The proportion of left and right arm predictions in the opaque condition. (E) The proportion of left and right arm predictions in the transparent condition. See also Videos S1, S2, and S3.

Is it more difficult to judge what a robot with non-human visual capability, as compared with human-like vision, knows about things that happen in the environment? Experiment 1 engaged with this question by subjecting experimental participants to a video of a humanoid robot responding to questions about the location of a ball (Figure 1A, left). The ball had been displaced while a curtain obstructed the robot's view. The robot responded to the location queries by using one of its arms to point toward one of two containers, one of which contained the ball. As participants watched the video, their anticipatory saccades toward the robot's arms were recorded as a measure of belief attribution. See the separate Transparent Methods section for the details of the methodology employed in this study. Note that all subsequent occurrences of the words “left” or “right,” as applied to robot arms and containers, refer to the perspective of the reader (not the robot).

In the *opaque condition* (*N* = 30, mean age = 25 years, 37% female) the robot was asked to point out the correct location of the ball despite the presence of an opaque blackout curtain that obstructed the robot's view while the ball was displaced (Figure 1A, middle, see also Video S1). Since it would be impossible for a human to see through the curtain in this scenario, repeatedly succeeding in this task without reliance on extraneous sources of information can be considered as requiring superhuman visual capability. If a majority of the participants in the opaque condition predict that the robot will point toward the left basket (where the ball was located before it was displaced), this reflects a tendency toward assuming that the robot is unable to see through the curtain that is opaque to humans (human-like visual capability). If they predict that the robot will point toward the right basket (where the ball was located after it was displaced), they are likely to assume the robot is able to see through the curtain that is opaque for humans (superhuman visual capability).

Video S1. Test Trial in the Opaque Condition, Related to Figure 1

In the *transparent condition* (*N* = 30, mean age = 25, 40% female), the robot was asked to point out the correct location of the ball in the presence of a fully transparent shower curtain obstructing the robot's view of the scene (Figure 1A, right; Video S2). Since a human would be able to see through the curtain in this scenario, repeatedly failing this task without reliance on extraneous sources of information can be considered as indicative of subhuman visual capability. If a majority of the participants in the transparent condition predict that the robot will point toward the left basket, this reflects a tendency toward assuming that the robot is unable to see through a structure that is transparent to humans (subhuman visual capability). If they predict that the robot will point toward the right basket, they are likely to assume the robot to be able to see through the transparent curtain (human-like visual capability).

Video S2. Test Trial in the Transparent Condition, Related to Figure 1

Participants in both conditions were subjected to a familiarization phase before the test trial (Video S3). Figure 1B provides an overview of the logic of the experiment.

Video S3. Familiarization Trials in the Opaque and Transparent Conditions, Related to Figure 1

#### Results

The proportion of anticipatory saccades toward the right robot arm in the *second familiarization trial* (*N* = 60) was 20 of 22 (91%; Figure 1C; i.e., 22 of the 60 participants exhibited anticipatory saccades toward any of the arms). This indicates a tendency among participants toward assuming that the robot is able to respond in a goal-directed manner to the location queries (p < .0005, *g* = .41, two choice binomial test, two tailed). The proportion did not statistically significantly differ between the two conditions (p = .481, Fisher's exact test, two tailed). It can therefore be concluded that participants assumed the robot to be capable of seeing the placement of the ball and responding appropriately to the location query. Hence, the anticipatory measure was sensitive to participants' assumptions about the robot's visual capability.

In the *opaque condition* (*N* = 30), the proportion of anticipatory saccades toward the left robot arm was 11 of 13 (85%; Figure 1D). This indicates a tendency toward the assumption that the robot was unable to see the displacement of the ball through the opaque curtain (p = .022, *g* = .35, two choice binomial test, two tailed). If the robot in the experiment—contrary to the expectations of most participants—in fact had the capability to see through the opaque curtain, then a majority of participants' predictions would have been incorrect. This suggests that it may be more difficult to predict the behavior of a previously unmet robot with non-human vision.

In the *transparent condition* (*N* = 30), the proportion of anticipatory saccades toward the left robot arm was 4 of 10 (40%; Figure 1E). This indicates the absence of a tendency toward assuming that the robot was able (or unable) to see the change-of-location event through the opaque curtain (p = .754, *g* = .10, two choice binomial test, two tailed). This means that participants could only predict the behavior of the robot approximately at the level of chance, suggesting a high level of uncertainty regarding the visual capabilities of the robot.

#### Discussion

We found that a majority of participants in the opaque condition assumed that the robot could not see through the curtain that is opaque to humans. The robot in the videos had no actual visual capabilities (see Transparent Methods section). However, if the robot in fact had been able to see through the curtain, the majority of their predictions would have been incorrect. This suggests that it might be more difficult to predict the behavior of robots whose visual capabilities differ from humans, particularly in first encounters. We also found that there was no clear tendency among participants in the transparent condition to assume that the robot could or could not see through the curtain that was transparent to humans. This means that if the robot in fact had an active perceptual system, then only half of the participants' predictions would have been correct.

### Experiment 2: Auditory Belief Tracking

For our second experiment, we wanted to complement our preceding investigation of visual belief tracking by asking whether it is more difficult to judge what a robot with non-human auditory capability as compared with human hearing knows about the environment. We also wanted to assess the strength of people's assumptions that robots have similar perceptual capabilities to themselves by investigating whether people would attribute a relatively advanced human perceptual capability to a robot.

**Figure 2:**
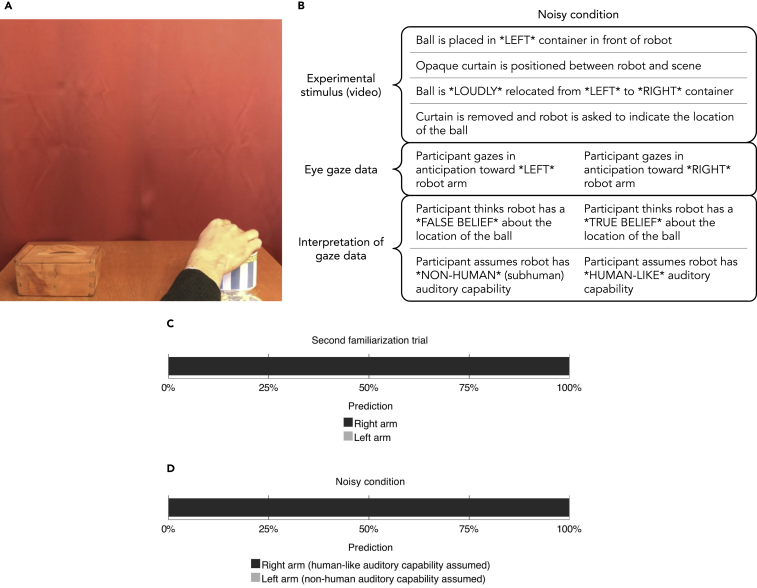
Auditory Belief-Tracking Experiment (A) Noisy containers. (B) Overview of the logic of the experiment. ∗LEFT∗ and ∗RIGHT∗ refer to baskets and robot arms from the reader's perspective. (C) The proportion of left and right arm predictions in the second familiarization trial. (D) The proportion of left and right arm predictions. See also Videos S4 and S5.

To test this, we designed an altered version of the stimulus used in the opaque condition in the previous experiment, where the robot—if it were a human person—would be aware of the relocation of the ball despite being separated from the scene by the opaque blackout curtain. In this new condition, which we called the *noisy condition* (*N* = 30, mean age = 25, 20% female), participants' ability to track the belief of the robot about the location of the ball depended on their estimation of the robot's auditory capability. As in the opaque condition, participants watched as the blackout curtain was pulled in front of the robot and the experimenter changed the location of the ball. However, in this condition the containers made distinctive sounds as the experimenter opened and closed their respective lids and dropped the ball into them (Figure 2A, Video S4). The sounds were such that they would instantly give away (to a person) the location of the ball every time it was moved from one container to the other. That is, a person would (after being exposed to the sounds during the familiarization phase, see Video S5) be aware of the relocation of the ball in virtue of being able to recognize the sounds *as* sounds that the containers make when interacted with. Note that this is a relatively advanced feat that requires not only registering sound but also associating different types of sensory stimuli (auditory and visual impressions of container-and-ball interactions) and, crucially, understanding the relevance of this association to the task at hand. Figure 2B provides an overview of the logic of the experiment.

Video S4. Test Trial in the Noisy Condition, Related to Figure 2

Video S5. Familiarization Trials in the Noisy Condition, Related to Figure 2

If a majority of the participants in the noisy condition predicts that the robot will point toward the left container (where the ball was located before it was displaced), this reflects a tendency toward assuming that the robot is unable to hear the displacement of the ball (non-human auditory capability). If they predict that the robot will point toward the right container (i.e., where the ball was located after it was displaced), they assume the robot is endowed with this relatively sophisticated human (-like) capability.

#### Results

The proportion of anticipatory saccades toward the right robot arm in the *second familiarization trial* (*N* = 30) was 12 of 12 (100%; Figure 2C). This indicates a tendency among participants toward assuming that the robot is able to respond in a goal-directed manner to location queries (p < .0005, *g* = .5, two choice binomial test, two tailed). It can therefore be concluded that participants assumed the robot to be capable of seeing the placement of the ball and responding appropriately to the location query. Hence, the anticipatory measure was sensitive to participants' assumptions about the robot's visual capability.

The proportion of anticipatory saccades toward the left robot arm was 8 of 8 (100%; Figure 2D). This indicates a tendency toward the assumption that the robot was able to hear the displacement of the ball (p = .008, g = .5, two choice binomial test, two tailed). If the robot in the experiment—contrary to the expectations of all participants—in fact did not have the capability to hear the displacement of the ball, then all of the participants' predictions would have been incorrect.

#### Discussion

We found that a majority of participants assumed that the robot could hear the displacement of the ball taking place on the other side of the opaque curtain. If the robot had in fact not been able to hear the displacement of the ball, the majority of their predictions would have been incorrect. This suggests that it might be more difficult, in first encounters, to predict the behavior of robots whose auditory capabilities differ from humans. Furthermore, the finding that participants were willing to ascribe this capability to the robot indicates that their anthropocentric assumptions were strong.

### Experiment 3: Endogenous Adaptation

Are people able to resolve their uncertainties about the perceptual capabilities of robots based on observing them interact with their environment? As a continuation of the previous experiments, we observed how the predictions of participants in the opaque and transparent conditions were affected by subsequent prolonged exposure to the robot's actions. That means, participants were given the opportunity to revise their assumptions about the robots' visual capabilities based on contradicting evidence from observation. Participants in the opaque condition observed the robot correctly pointing out the location of the ball despite the intervening opaque curtain and participants in the transparent condition observed the robot not being able to do so despite the transparency of the intervening curtain.

To test whether participants were able to resolve their incorrect assumptions about the robots' visual capabilities based on observing how they acted in response to the location queries, we measured their ability to track the beliefs of the robot over the course of a number of consecutive action anticipation trials, where the object displacement altered from left-to-right basket in the first trial, right-to-left basket in the second trial, left-to-right basket in the third trial, and so on, for a total of five trials.

#### Results

**Figure 3:**
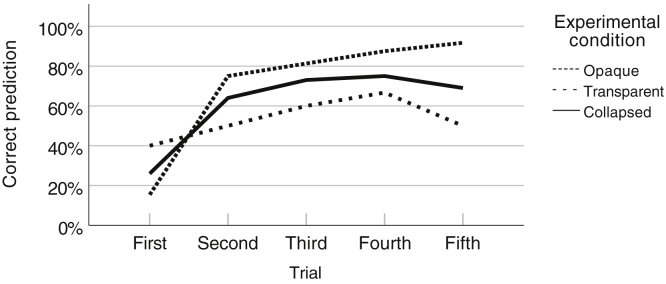
Endogenous Facilitation Experiment The proportion of correct predictions across consecutive test trials.

A Cochran-Armitage test of trend was run to determine whether a linear trend exists between the number of observed robot actions and participants' ability to correctly predict the robots' actions. The proportion of correct predictions in the first to fifth action-anticipation trial (and the number of predictions in each trial) was .26 (*n* = 23), .64 (*n* = 22), .73 (*n* = 26), .75 (*n* = 20), and .69 (*n* = 26), respectively. The Cochran-Armitage test of trend showed a statistically significant linear trend between the number of observed robot actions and the ability to correctly predict the robot's actions, p = .003, with higher numbers of observed robot actions associated with a higher proportion of correct belief-sensitive predictions of the robots' actions (Figure 3).

#### Discussion

We found that repeated observations of the robots' actions had a positive effect on participants' ability to track the robots' perceptual beliefs. This suggests that people can revise (incorrect) anthropocentric assumptions about the perceptual capabilities of robots endogenously, based on observing them interact with the environment. This is promising for longitudinal human-robot interactions where there is opportunity for people to familiarize themselves with the capabilities of a robot. However, this ability may not come into effect in first-time encounters with robots or interactions where there is little opportunity to observe how robots interact with the environment. External aids are therefore likely needed as a partial solution to the problem of inferring the perceptual beliefs of robots with different-from-human perception.

### Experiment 4: Exogenous Facilitation

Can inferring a robot's perceptual beliefs be facilitated by providing people with verbal information about its non-human perceptual capability? To test this, we set up an experiment based on the conditions in the previous experiments (opaque, transparent, and noisy). Two independent groups of participants were subjected to three consecutive action-anticipation test trials: first an “opaque” trial (Video S1), followed by a “transparent” trial (Video S2), and finally a “noisy” trial (Video S4). Participants in one group received on-screen verbal information about the robots' perceptual capabilities (*N* = 32, mean age = 26, 35% female) prior to each trial. Participants in the other group (*N* = 33, mean age = 24, 32% female) received no such information. Participants in both conditions were subjected to a familiarization phase before the test trial (Video S3). The information provided prior to each trial was as follows:

• Opaque: “The robot you are about to see CAN see through structures that are non-transparent to humans.”
• Transparent: “The robot you are about to see CANNOT see through structures that are transparent to humans.”
• Noisy: “The robot you are about to see CANNOT identify the location of objects based on sound. Also, the robot CANNOT see through structures that are opaque to humans.”

#### Results

**Figure 4:**
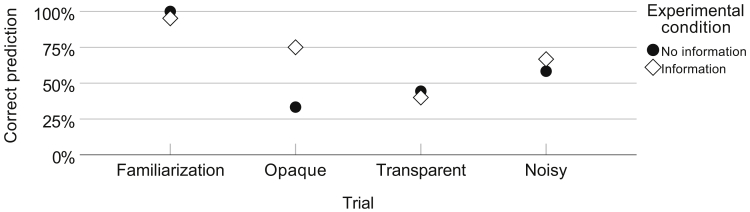
Exogenous Facilitation Experiment The proportion of correct predictions across opaque, transparent, and noisy test trials in the no-information and information conditions.

A chi-square test of homogeneity was run to assess whether there was a statistically significant difference between the binomial proportions (correct versus incorrect prediction) in the two independent groups (information versus no information) across the three action anticipation test trials. The proportion of correct prediction was 24 of 38 (63%) in the instructions group and 16 of 36 (44%) in the no-instructions group, a statistically non-significant difference in proportions of .19, p = .106, ϕ= .32 (Figure 4). There was a statically significant effect of providing information in the opaque trial, with a difference in proportions of .42, p = .020, ϕ= .42. No statistically significant differences were found in the other trials.

#### Discussion

We did not find an overall effect of providing verbal information about the perceptual capabilities of robots to experimental participants on their ability to infer the perceptual beliefs of the robots. This indicates that providing verbal information about the perceptual capabilities of robots prior to first encounters might be of limited value as a tool for facilitating interaction with robots with different-from-human perception. Possibly, participants' anthropocentric assumptions about the robots' perceptual capabilities were strong enough to resist revision based on the provided information. Future research should empirically assess other types exogenous solutions to the problem of inferring the perceptual belief of robots that do not rely on verbal information (for a list of suggested candidate solutions, see Thellman and Ziemke, n.d., submitted).

## Discussion

The research reported here was motivated by the conjecture that people's uncertainties about the perceptual capabilities of robots make it difficult to infer their perceptual beliefs (i.e., what robots know or believe about the environment based on their perception). We refer to this as the *perceptual belief problem in HRI* (Thellman and Ziemke, n.d., submitted). In the present study, we tested this conjecture by subjecting experimental participants to an anticipatory-looking false-belief task where they had to reason about a robot's perceptual beliefs in order to predict its behavior. We found that a majority of the participants in our experiments predicted the robots' actions based on *anthropocentric assumptions* about their visual and auditory capabilities. Expecting humanoid robots to act as humans can be considered as reasonable in circumstances where one has no prior knowledge about its capabilities. However, it is important to note that robots (even those that to some degree look like humans) will not, in the foreseeable future, be able to perceive and act as humans typically do in many situations, especially, as we have previously argued, in interactive situations where appropriate action presupposes human-like capabilities (Thellman and Ziemke, n.d., submitted). Hence, it can *also* be reasonable to expect robots to have different perceptual capabilities than humans, such as the “superhuman” ability to see through objects that are opaque to humans (experiment 1) or the “subhuman” inability of not being able to infer the presence of an object from associated sound cues (experiment 2). Because the capabilities of robots are diverse and usually cannot be directly inferred based on appearance, it is difficult for people to know *when* to expect robots to have different-from-human capabilities and *which* specific capabilities to expect. As demonstrated in our experiments, this can negatively affect people's ability to predict robot behavior in interactions with robots. The results from the first pair of experiments in our study, particularly the finding that people's anthropocentric assumptions are rather strong (experiment 2), strengthens our conviction that the perceptual belief problem is a substantial problem in HRI, especially in first encounters with robots where there is little opportunity to familiarize with a robot's capabilities. It is also worth noting that the problem might become increasingly relevant in the future as robotic technology develops, allowing robots to be endowed with perceptual capabilities that are more diverse and more different from human capabilities.

In the second pair of experiments, we explored two different types of “solutions” to the perceptual belief problem. Experiment 3 considered whether people are able to revise their incorrect assumptions about the perceptual capabilities of a robot based on observing it interact with the environment and thereby improve their chances of correctly predicting its actions. We found this to be the case; participants' predictions were increasingly correct as they made a number of observations of the robots' actions. This indicates that people might be able to adapt or “tune in” to the unique capabilities of a robot endogenously if given opportunity to observe and/or interact with it over an extended period of time. Exogenous solutions might still be necessary to solve the perceptual belief problem, especially in first encounters with robots, and might serve as a compliment to endogenous adaptation in longitudinal human-robot interactions. In experiment 4, we assessed the efficacy of one type of exogenous solution: facilitating inferences of a robot's perceptual beliefs by providing verbal information about its perceptual capability prior to the first encounter. We found this to be an inefficient tool for improving participants' belief-sensitive predictions of the behavior of the robots in our experimental setup. However, there are other types of potential exogenous solutions to the perceptual belief problem (e.g., “white-cane solutions, Thellman and Ziemke, n.d., submitted) that might work better but have not yet been systematically evaluated.

In conclusion, we have presented the first systematic study specifically concerned with people's ability to infer the perceptual beliefs of robots (i.e., what robots know or believe about the environment based on their perception). Based on the results from this study we draw three general conclusions. First, we saw a tendency among participants in our experiments to predict the behavior of previously unmet robots based on anthropocentric assumptions that they have similar (human-like) perceptual capabilities to themselves. We take this as evidence that it is more difficult for people to track the perceptual beliefs of robots whose perceptual capabilities significantly differ from human perception, especially in circumstances where there is little opportunity to familiarize with the robot's capabilities. Second, we found that the ability to predict robot behavior improved with prolonged exposure. From this we conclude that people can endogenously adapt or “tune in” to the unique (non-human) perceptual capabilities of robots. Finally, we tested the efficacy of providing verbal information about the perceptual capabilities of robots as a way to facilitate inference of its perceptual beliefs. Finding no significant effect, we conclude that solutions that rely on verbal information might not necessarily be helpful.

Taken together, the above findings demonstrate that the perceptual belief problem is a substantial problem in HRI. To the extent that non-human perceptual capabilities are useful for navigating the physical world, equipping robots such capabilities can be expected to become increasingly common. It may turn out, however, that equipping robots with non-human perception makes it more difficult for people to interpret robots and to interact with them.

### Limitations of the Study

The present study has a number of limitations. Owing to the lack of previous application of the experimental paradigm used in the study (anticipatory-looking false-belief task) within the domain of human-robot interaction, and, to some extent on members of the adult population, we chose a slightly larger sample size than previous studies employing the paradigm (e.g., Senju et al., 2009; Southgate et al., 2007). We originally estimated that a sample size of 30 would support the detection of a medium effect size (Cohen's *g* = .25) at the .05 α level at 1-β = 0.8. However, data analysis revealed that the number of anticipatory gazes recorded in each test trial relative to the number of participants in each trial was 38% (this number was relatively stable across all test trials in the study, *Min* = 27%, *Max* = 53%, *SD* = 8%). The median cell value was 12 (*Min* = 8, *Max* = 16, *IQR* = 3.5). Although the obtained cell value is comparable with previous studies, the number of anticipatory saccades per participant and test trial was considerably lower in our study. This could potentially be attributed to the study context (judgments of robot behavior) and/or the adult participant sample. Although the obtained data meets the assumptions of the statistical tools that were used for analysis, future studies that employ similar anticipatory gaze measures might benefit from increased sample sizes.

Despite the relatively small number of anticipatory saccades per participant in each trial obtained, the sample sizes in our experiment were sufficient to detect large effect sizes (*g* > .25) at α = .05 in the binomial tests conducted on data obtained from the opaque and noisy conditions, including familiarization trials, at a power greater than 0.8. This means that these results are unlikely to be due to lack of statistical power. To check whether the non-significant results in the transparent condition (10 recorded anticipatory gazes) were due to lack of statistical power, we conducted power analysis with 1-β set at 0.8, α = .05, two tailed. We used the average effect size obtained from the two other similar test conditions (opaque and noisy) to calculate an expected effect size, *g* = 0.455 (very large). The analysis showed that the estimated sample size required to meet these criteria is nine. However, given the obtained number of data points we would not have been able to detect a small or medium effect (*g* < .25) at the level of β = 0.8 as this would require at least 30 data points. This means that the statistically non-significant results in the transparent condition could have been due to lack of statistical power.

We have drawn general conclusions about people's inferences of the perceptual beliefs and capabilities of robots based on the results of the study. We would like to address three issues of generalizability:

• The study includes only a specific type of humanoid robot (Pepper). Do the results generalize to *all* robots?
• The study includes an idiosyncratic sample of experimental participants. Do the results generalize to *all* people?
• The video-based paradigm is void of actual interaction with robots. Do the results generalize to real-life interactions with robots?

Owing to the significant diversity of people and robots, naturally no single HRI study can support general claims about interactions with robots with absolute certainty. The short answer to the three questions above is therefore that we do not know. Nevertheless, we think that there is evidence to support the assumption that the results, by large, generalize in all three cases. A large body of theory-of-mind research, including a meta-analysis of two decades worth of false-belief experimentation (Wellman et al., 2001), supports the following general conclusions about human belief inference that we think are relevant to the issues of generalizability stated above:

1) People's ability to infer the beliefs of an agent is largely independent of its nature of the protagonist, that is, whether the agents is, for example, a puppet or doll, a pictured character, a videotaped person, or a real person present in, or absent from, the current situation (Wellman et al., 2001).
2) The capacity for belief inference is limited in children but stabilizes in adulthood (Wellman et al., 2001), with the exception of individuals with certain pathological conditions such as autism (Baron-Cohen, 1997) and schizophrenia (Bora et al., 2009).

We think it is justified, based on (1), to assume that the results of the present study, by large, generalize to different types of robots. Although perceptual capabilities are not directly observable, a robot's external appearance can give rise to expectations about its internal capabilities (Goetz et al., 2003) and therefore it is relevant to consider whether the results generalize, for example, to non-human-looking robots. However, we presently see little reason to expect the ability to infer the perceptual capabilities of robots based on their actions *not to* extend to all types of robots. We plan to extend our investigations to include the interaction between human-like robot appearance and inferences of internal states and capabilities in future work to test this.

Furthermore, we think that (1) justifies the assumption that the results, by large, generalize to actual interactions with robots, in particular first encounters with robots. People are in some cases able to resolve their uncertainties about robots by interacting with them, in what could perhaps be described as in an “experimental” manner, to, for example, see how they respond to different stimuli or by asking directly them about their capabilities. Furthermore, previous research indicates that social interaction studies that employ experimental methodology (e.g., video-based paradigms such as ours) may have limited ecological value (Henschel et al., 2020; Krishnan-Barman et al., 2017). We chose the present paradigm mainly because of the experimental control it affords, and also on the grounds that the above-cited theory-of-mind research has found that the nature of the agent (e.g., video presence or real-life presence) plays little or no role in belief inference. We can speculate, based on such findings, that our results generalize to real-life interactions, especially in time-critical encounters where people rely on observation only. However, we cannot conclusively predict this without further empirical evidence.

Finally, we think that it is reasonable to assume, on the basis of (2), that the ability to infer the perceptual beliefs of robots to follows a similar developmental and pathological pattern, and that our results therefore, by large, generalize to members of the adult population.

### Resource Availability

#### Lead Contact

Further information and requests for resources should be directed to and will be fulfilled by the Lead Contact, Tom Ziemke (tom.ziemke@liu.se).

#### Materials Availability

This study did not generate any new unique reagents.

#### Data and Code Availability

The original data presented in this paper are available at Mendeley Data: https://doi.org/10.17632/4gvv343b5x.1.

## Methods

All methods can be found in the accompanying Transparent Methods supplemental file.

## Supplemental Information

Document S1. Transparent Methods and Figures S1–S5
