# Supplementary material for: Do You See what I See? Tracking the Perceptual Beliefs of Robots
Source: iScience. 2020 Sep 29;23(10):101625. doi: 10.1016/j.isci.2020.101625 (PMC7567989; doi:10.1016/j.isci.2020.101625)
Supplement: Document S1. Transparent Methods and Figures S1–S5 [file mmc1.pdf]

iScience, Volume *23*

Supplemental Information

Do You See what I See?

Tracking the Perceptual Beliefs of Robots

Sam Thellman and Tom Ziemke

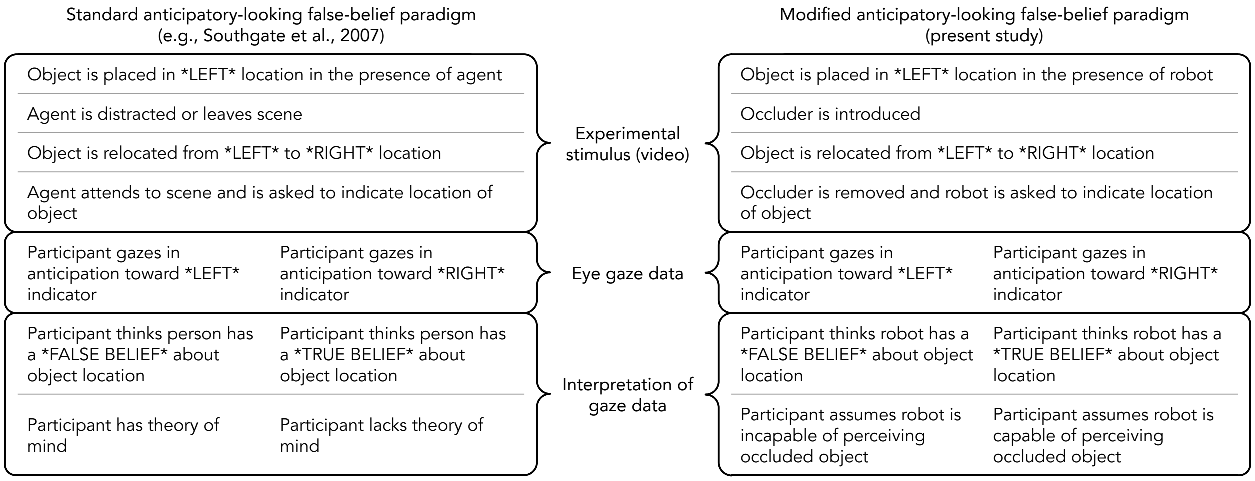

Figure S1. Overview of modified experimental paradigm used in the present study (right) and its source (left). *LEFT* and *RIGHT* refer to locations and robot arms from the perspective of the participant in the experiment. Related to Figure 1.

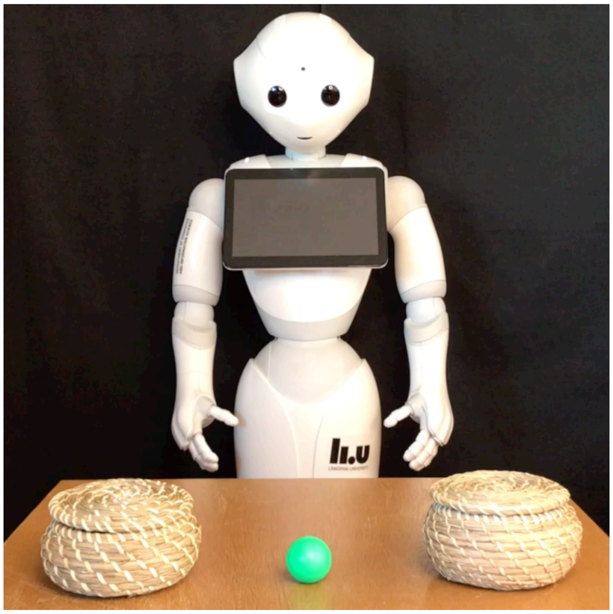

Figure S2. Scene from video stimuli. Related to Figure 1.

Focus	Experiment	Conditions (*N*)	Research question
Problem	1: Visual belief tracking	Opaque (30), Transparent (30)	Is it more difficult to judge what a robot with non-human as compared to human-like vision knows about the environment?
	2: Auditive belief tracking	Noisy (30)	Is it more difficult to judge what a robot with non-human as compared to human-like hearing knows about the environment?
Solution	3: Endogenous adaptation	Opaque (30), Transparent (30)	Are people able to learn and adapt to the non-human perception of a robot by observing the robot interact with the environment?
	4: Exogenous facilitation	No information (33), Information (32)	Can inferring a robot's perceptual beliefs be facilitated by providing people with verbal information about its non-human perceptual capability?

Figure S3. Study design overview. Note that some of the independent conditions feature in multiple experiments. Related to Figure 1.

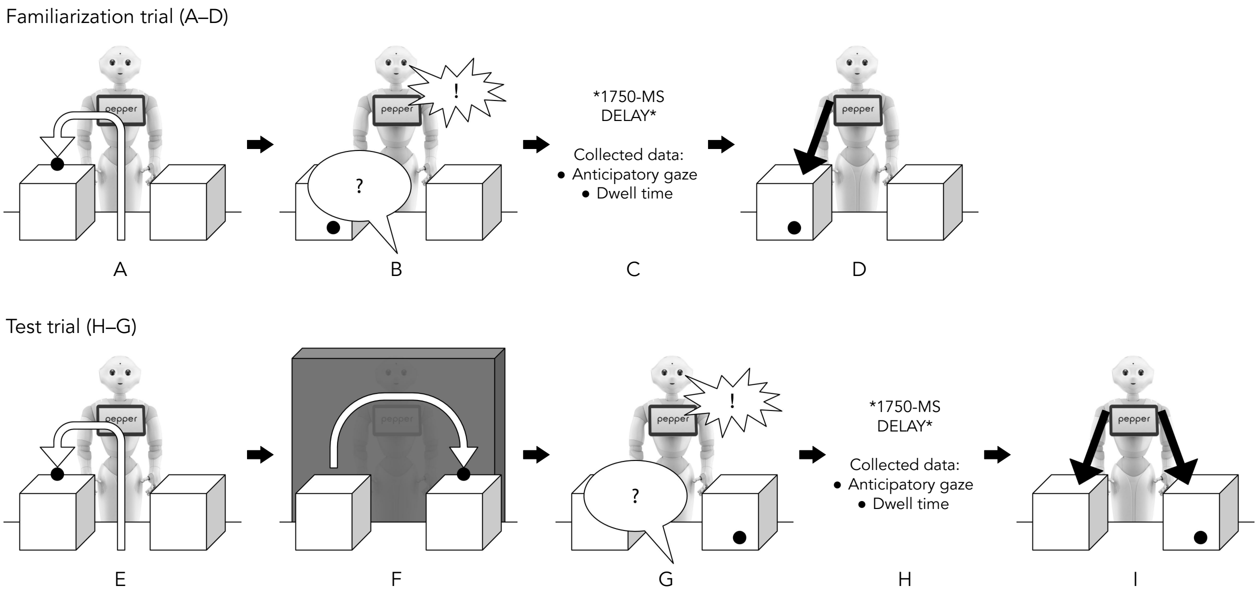

Figure S4. Sequence of events depicted in video stimulus presented to participants. Empty arrows indicate movements of the hand manipulating the ball and containers. Solid arrows indicate the robot's pointing gestures. Eye-gaze data was collected at points C and H. Related to Figure 1.

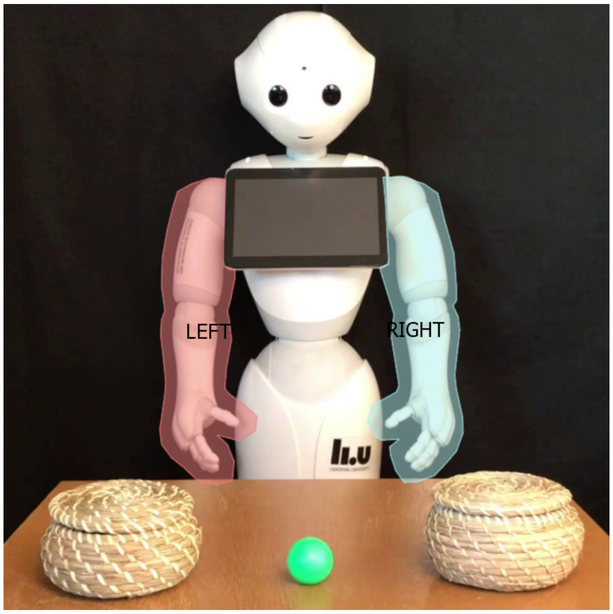

Figure S5. Areas of interest used to evaluate the target of participants' anticipatory saccades. Related to Figure 1.

Transparent Methods

The reported study investigated people's ability to track the perceptual beliefs of robots (i.e., what robots know or believe about the environment based on their perception) by subjecting experimental participants to a false-belief task where they had to reason about a robot's perceptual capability. The task was based on the standard "change-of-location" false-belief task paradigm employed in *theory of mind* research (e.g., Baron-Cohen, Leslie and Frith, 1985; Wimmer and Perner, 1983). When faced with a standard false-belief task, an experimental participant is made aware that an agent has a false belief about some state of affairs (e.g., by showing that an object that belongs to the agent is displaced in its absence) and is then asked to predict how the agent will behave given its false belief (e.g., by asking where the agent will look for the object when returning to retrieve it).

The standard false-belief task has been used previously to study belief attribution to robots (e.g., Banks, 2019). However, it does not accommodate studying attributions of *perceptual* beliefs (i.e., beliefs that are the output of an agent's perceptual system). The protagonist or "false believer" in standard false-belief task scenarios is typically depicted as being absent or distracted from the event (e.g., displacement of object) which leads to the false belief. This renders considerations about the agent's perceptual capability irrelevant to the task at hand. Again, the purpose of the present study was to investigate people's ability to infer the perceptual beliefs of robots based on their assumptions about their perceptual capabilities. We therefore modified the standard false-belief task stimulus to accommodate such considerations in the belief attribution process (see Figure S1). In our modified version, the agent's belief about the relevant state of affairs is instead made contingent upon its ability to perceive the change-of-location event through opaque or transparent curtains. As a consequence of this, participants subjected to our version of the task has to judge the agent's – in our case the robot's – belief based on their assumptions about its perceptual capability.

The study utilized the anticipatory-looking (AL) false-belief task paradigm (Clements and Perner, 1994; Schneider, Bayliss, Becker and Dux, 2012; Senju, Southgate, White and Frith, 2009; Southgate, Senju and Csibra, 2007) to measure participants' *implicit* tracking of the robot's beliefs. The AL paradigm is based on the standard change-of-location false-belief task but capitalizes on participants' spontaneous looking behavior instead of explicit, verbal responses to questions such as "Where does the agent think the object is?" or "Where will the agent look for the object?" (Schneider, Slaughter and Dux, 2017). Implicit false-belief tasks avoid some of the methodological issues that derive from the fact that verbal responses are subject to rationalization and are not always indicative of a person's behavior in social interactions (Gawronski and Bodenhausen, 2006; Greenwald and Banaji, 1995). We have argued elsewhere that this property of verbal measures might be particularly troublesome in the context of human-robot interaction studies, where people's answers to questions about the minds of robots have been found to vary significantly depending on a number of factors that are difficult to control for experimentally (Thellman and Ziemke, 2019; Thellman and Ziemke, submitted).

In our modified anticipatory-looking false-belief task paradigm, the eye-gaze pattern of experimental participants was recorded as they watched a video of a robot answering simple object location queries ("Pepper, where is the ball?") by pointing toward one of two opaque baskets with lids which was either empty or contained a ball (Figure S2). The queries triggered anticipatory gazes toward the robot's arms which can be interpreted as driven by participants' estimations of the robot's belief about the location of the ball. These estimations, in turn, depended on their assumptions about the perceptual capability of the robot (i.e., whether the robot was capable of perceiving the relocation of the ball from one of the

baskets to the other in the presence of an occluding curtain). Using this experimental paradigm, we were able to investigate how participants' judgment of the robot's (perceptual) belief about the location of the ball varied as a function of:

- whether the robot's task of pointing out the location of the ball was possible or impossible assuming human or non-human vision (experiment 1) or hearing (experiment 2),
- prolonged exposure to the robot's responses to the object location queries (experiment 3),
- providing information about the perceptual capability of the robot prior to exposure (experiment 4).

All experiments in this study were based on the paradigm described above and utilized the same general procedure, equipment, and measures (described in the remainder of this section of the paper). Experimental conditions and stimuli differed between experiments. We describe these aspects of the study separately in the Results section of the main paper. See Figure S3 for an overview of the study design.

Participants and Procedure

University students with different curricula, including programming, statistics, chemistry, economics, and cognitive science, were asked to participate in a research study in which their gaze behavior would be recorded as they watched a video of a robot interacting with its environment. A total of 155 participants accepted and were randomly assigned to one of the five independent experimental conditions in the study. The conditions differed in the video stimulus presented to participants.

After agreeing to participate in the study, each participant was shown to the designated experiment location (a distraction-free room) and was briefly introduced to the practical setup of the experiment, focusing on the eye-tracking equipment in particular. After the introduction, each participant was seated in appropriate proximity from the eye- tracking monitor and was subjected to a brief (10 sec) eye-tracking equipment calibration procedure. The calibration procedure was repeated, if necessary, until acceptable calibration was achieved. Finally, the participant was fitted with headphones and the video started playing. Because of the implicit nature of the anticipatory-looking measure used in the study, no explicit instructions were given to participants. The study was carried out in accordance with institutional guidelines, with written informed consent from all participants in accordance with the Declaration of Helsinki.

Experimental Stimuli

The stimuli used in the study were short video clips of a robot asked to point to the location of a ball placed in one of two baskets. All videos followed the same structural pattern (differences are described separately in the results section of the main paper). Each video was divided into two phases: a familiarization phase consisting of two consecutive familiarization trials, and a test phase consisting of a number of consecutive test trials.

In the two familiarization trials, a ball was placed in one of the baskets by the hands of a person located at the camera's point of view (empty arrow in Figure S4A). A voice (appearing to belong to the person manipulating the ball and baskets) then sounded: *Pepper, where is the ball?* (Figure S4B). A few seconds later, a sound from the robot ("blip!") and flash of color in the robot's eyes appeared simultaneously. From the onset of this signal, there was a 1750 millisecond time frame (Figure S4C) directly followed by a pointing gesture by the robot toward the basket containing the ball (Figure S4D). In the first

familiarization trial the ball was placed in the left basket; in the second trial it was placed in the right basket. The purpose of the familiarization phase was twofold. Firstly, it conveyed to participants that the robot was able to act in accordance with the goal to locate and point toward the basket containing the ball. Secondly, the second familiarization trial functioned as a validation check, indicating whether the experimental stimuli is able to triggered anticipatory gaze behavior. The familiarization phase transitioned directly into the test phase without pause or notification.

Each test trial proceeded as follows. A ball was placed in one of the baskets (Figure S4E). A curtain was pulled into the scene, between the robot and the objects, and the ball was removed from the basket and put into to the other basket (Figure S4F). Following object relocation, the curtain was pulled away and a voice (same as in prior trials) sounded: *Pepper, where is the ball?* (Figure S4G). A few seconds later, a sound from the robot ("blip!") and flash of color in the robot's eyes appeared simultaneously. From the onset of this signal, there was a 1750-ms time frame (Figure S4H) directly followed by a pointing gesture by the robot toward one of the baskets (Figure S4I). The three videos that featured in the conditions in experiments 1–3 varied in length between 03:45 and 04:19 (min:sec). This variation was partly due to a small intended variation in the pause between the location query (*Pepper, where is the ball?)* and the query confirmation signal (sound and eye flash), and inadvertent variation in the time it took to relocate the ball between baskets in each trial. The video in the two conditions in experiment 4 was 1:49. The differences between the videos are described separately in the results section of the main paper.

Equipment and Measures

The robot featured in the videos was a SoftBank Robotics Pepper V1.6 running NAOqi 2.5. The robot's behavior was fully scripted for the purposes of stimuli production. Participant gaze behavior was recorded at 30 frames per second using a SensoMotoric Instruments (SMI) RED500 remote eye-tracking device integrated to a 22-inch monitor. Stimuli were presented to participants using SMI's iView X 2.8 and Experiment Center 3.6 software applications. Analysis was conducted using SMI BeGaze 3.6 and IBM SPSS Statistics 24.

As an implicit measure of belief tracking, the target (left or right robot arm) of each participant's first saccade during the 1750-ms time frame (after signal onset and before robot pointing gesture) was coded after carefully inspecting each participant's gaze replay file in slow motion. Areas of interest were defined around the arms of the robot so as to minimize overlap with the rest of the robot's body and objects in the scene and with some padding to accommodate eye-tracking inaccuracy, in line with recommendations in Holmqvist et al. (2011) (Figure S5). All p-values reported in the paper were two-tailed. Effect sizes were calculated using Cohen's g for one-sample binomial proportions and Phi (φ) for two-sample binomial and ordinal data. According to Cohen (2013), g (and φ) effect size magnitudes can be interpreted as 0.10 < 0.15 (0.1) being a small effect size, 0.20 < 0.25 (0.3) being a medium effect size, and < 25 (0.5) being a large effect size. There were no main effects of gender or age in any of the experiments, so we collapsed across gender and age in all reported analyses.

Supplemental References

Banks, J. (2019). Theory of mind in social robots: Replication of five established human tests. International Journal of Social Robotics 12, 403–414.

Baron-Cohen, S., Leslie, A. M. and Frith, U. (1985). Does the autistic child have a "theory of mind"? Cognition 21, 37–46.

Clements, W. A. and Perner, J. (1994). Implicit understanding of belief. Cognitive Development 9, 377–395.

Cohen, J. (2013). Statistical Power Analysis for the Behavioral Sciences (Academic Press).

Faul, F., Erdfelder, E., Lang, A. G., and Buchner, A. (2007). G* Power 3: A flexible statistical power analysis program for the social, behavioral, and biomedical sciences. Behavior research methods 39, 175–191.

Gawronski, B. and Bodenhausen, G. V. (2006). Associative and propositional processes in evaluation: an integrative review of implicit and explicit attitude change. Psychological Bulletin 132, 692–731.

Greenwald, A. G. and Banaji, M. R. (1995). Implicit social cognition: attitudes, self-esteem, and stereotypes. Psychological Review 102, 4–27.

Holmqvist, K., Nyström, M., Andersson, R., Dewhurst, R., Jarodzka, H. and Van de Weijer, J. (2011). Eye Tracking: A Comprehensive Guide to Methods and Measures (Oxford University Press).

Schneider, D., Bayliss, A. P., Becker, S. I. and Dux, P. E. (2012). Eye movements reveal sustained implicit processing of others mental states. Journal of Experimental Psychology: General 141, 433–438.

Wimmer, H. and Perner, J. (1983). Beliefs about beliefs: Representation and constraining function of wrong beliefs in young children's understanding of deception'. Cognition 13. 103–128.

Thellman, S., Giagtzidou, A., Silvervarg, A. and Ziemke, T. (2020). An Implicit, Non-Verbal Measure of Belief Attribution to Robots. In Companion of the 2020 ACM/IEEE International Conference on Human-Robot Interaction (ACM), pp. 473–475.
